# Biological Characterization and Glucosinolate Degradation Mechanisms of *Bacillus subtilis* BSY82 in Rapeseed Meal

**DOI:** 10.1155/anu/3661772

**Published:** 2025-07-30

**Authors:** Hong-yu Lu, An-Ning Shi, Jun Wang, Li-Juan Liu, Jiang-Yi Tang, Ya-Jun Wang, Xue-Zhi Zhu, De-Feng Zhang, Li-Yan Liu, Qing Wang

**Affiliations:** ^1^Pearl River Fisheries Research Institute, Chinese Academy of Fishery Sciences, Key Laboratory of Fishery Drug Development, Ministry of Agriculture and Rural Areas, Guangdong Provincial, Key Laboratory of Aquatic Animal Immune Technology, Guangzhou 510380, China; ^2^Key Laboratory of Sichuan Province for Fishes Conservation and Utilization in the Upper Reaches of the Yangtze River, Neijiang Normal University, Neijiang 641100, China; ^3^Guangdong Yuehai Feeds Group Co., Ltd., Guangdong Aquatic Animal Feed Engineering Technology Research and Development Center, Zhanjiang 524017, China

**Keywords:** *Bacillus subtilis* strain BSY82, biological characterization, fermented, glucosinolate, rapeseed meal

## Abstract

Glucoside is the main antinutritional factor (ANF) of rapeseed meal. In the present study, a bacterium with high efficiency in degrading rapeseed glucoside was screened from pond water. The initial concentration of 1.0 × 10^7^ CFU/mL bacterial solution was added to rapeseed meal and fermented for 48 h with a degradation rate of glucoside and isothiocyanate of 67% and 54%, respectively. BSY82 was identified by PacBio third-generation sequencing, and its biology was analyzed. Results showed that BSY82 belongs to *Bacillus subtilis*. The total length of genome was 4,242,094 bp, among which 89% was coding genes (3,777,298 bp). No virulence factors were predicted, based on a comparative analysis of the strain's coding genes against known virulence factor databases. Functional analysis showed that BSY82 had the ability to produce myrosinase. The activity of myrosinase in the crude enzyme solution of the strain was 1.67 and 2.12 μmol/min by spectrophotometry and high-performance liquid chromatography (HPLC). The results indicated that BSY82 strain could be used as the optimal material in rapeseed meal fermentation detoxification process.

## 1. Introduction

Rapeseed meal is a by-product of rapeseed oil extraction, with a protein content of approximately 36%. It is the second largest source of plant protein supply in aquatic feed and has remarkable potential in soybean meal substitution [1]. However, the use of rapeseed meal is greatly limited by the antinutritional factors (ANFs), such as glucosinolates, tannins, and phytic acid [2]. Because of glucosinolates restriction, the maximum amount of rapeseed meal that can be added to the fish diet is usually 15%–25% [3]. To improve the feeding value of rapeseed meal, researchers have explored many methods to remove the ANFs in rapeseed meal, including physical detoxification, chemical detoxification, and microbial fermentation [4]. Gu et al. [5] found that pressure of ≥1.6 MPa for 180 s can effectively remove glucosinolates and their degradation products. Critical carbon dioxide extraction and cold-pressed hexane extraction significantly reduced the glucosinolates and neutral detergent fiber. However, these methods also have significant drawbacks, such as reduction in amino acid content [6], high cost, and environmental pollution [7].

Microbial fermentation is a mild and cheaper strategy for decomposing and utilizing the ANFs in rapeseed meal. Studies have shown that the key to fermentation process is bacterial strain selection. *Lactobacillus acidophilus* could degrade 23.69% of glucosinolates, and the bacterium in combination with *Bacillus subtilis* and *Saccharomyces cerevisiae* increased the degradation rate to 30.73% [8]. A microbial mixture comprising *Lactobacillus plantarum*, *Bacillus subtilis*, and *Aspergillus oryzae* (fungus) successfully degraded 91.36% of glucosinolates [9]. Furthermore, the mixture of *Bacillus natto*, *Bacillus subtilis*, and *Saccharomyces cerevisiae* could degrade the oxazolidinedione and isothiocyanate in canola meal at the rate of 71.16% and 80.25% respectively [10]. Although multistrain fermentation process is economically more effective than single-strain, drawbacks of the antagonistic effects between strains, high nutrient loss, and difficulty in controlling the fermentation process are also evident. Therefore, screening of individual strains with highly efficient, harmless, and fast-growing characteristics is required.

In the present research, a highly effective glucosinolate degradation strain was screened and identified. The effectiveness, safety, and antimicrobial drug susceptibility of the strain were studied. Moreover, the mechanism of glucosinolate degradation was explored. The results will widen the utilization of rapeseed meal.

## 2. Materials and Methods

### 2.1. Source of Strains and Screening Strains

A total of 192 *Bacillus* and *Lactobacillus* strains were isolated from a grass carp (*Ctenopharyngodon idella*) culture pond at the Pearl River Fisheries Research Institute (23°06′5549″N, 113°22′1326″E) in 2021 using a membrane filtration enrichment method according to Fan et al. [11]. Water samples were first filtered through 0.22 µm membranes to concentrate microbial biomass. The retentate was serially diluted (10^–1^–10^–6^) and spread on two enrichment media selected to favor key functional groups: de Man–Rogosa–Sharpe (MRS) agar for *Lactiplantibacillus*/*Lactobacillus spp*. and Luria–Bertani (LB) agar for *Bacillus spp*. Plates were incubated aerobically at 37°C for 48 h, a temperature and oxygen regime that promotes the growth of these genera while suppressing many slow-growing or strict-anaerobic taxa. As a result, the dominant culturable genera obtained under these conditions were *Bacillus* and *Lactiplantibacillus*. Individual colonies were purified by repeated streaking and preserved at −20°C in 20% (v/v) glycerol for further characterization. The 192 isolates were inoculated on LB medium and cultured to an OD_600_ = 0.5, at 37°C. The cultures were then diluted 1:100 times in a rapeseed meal-based screening medium prepared as follows: 100 g of rapeseed meal was added to 900 mL of distilled water, boiled for 10 min, and the filtrate was collected with 8 layers of gauze. The filtrate was centrifuged at 7500 rpm for 10 min, and the supernatant was adjusted to 1 L as the crude rapeseed meal extract. For the screening medium, 100 mL of the crude extract was mixed with peptone (10 g), NaCl (10 g), and agar (15 g), then brought to a final volume of 1 L with distilled water. The medium was sterilized at 121°C for 20 min and poured into plates. A sterile iron cylinder was used to punch circular wells 7 mm in diameter in the prepared agar plates. Then, 100 µL of each bacterial suspension was dispensed into each well, and the plates were incubated at 37°C for 48 h before measuring colony and halo diameters. Colonies surrounded by transparent halos were scored as positive in this preliminary assay. The clear zones indicate that a strain can degrade insoluble or turbidity-causing components of the rapeseed meal extract, reflecting a general ability to break down meal constituents. Importantly, this halo-forming assay is not specific to glucosinolate hydrolysis; rather, it serves as an initial screen for strains with broad rapeseed meal degradation capability. Strains identified as positive in this screen were subsequently subjected to targeted glucosinolate degradation tests. In those follow-up experiments, solid-state fermentation of rapeseed meal was performed using each strain, and glucosinolate removal was precisely quantified by high-performance liquid chromatography (HPLC).

### 2.2. Hemolytic Characteristic Analysis

The candidate strains were inoculated on sheep blood plates at 37°C for 24 h. Hemolytic rings appeared around the bacterial colonies and were considered to contain hemolytic activity [12].

### 2.3. Protease and Cellulase Activities

In addition to glucosinolate removal, enhancing proteolysis and fiber degradation can further improve the nutritional value of fermented rapeseed meal; therefore, protease and cellulase activities of the isolates were also evaluated. To evaluate the ability of the screened isolates to produce extracellular enzymes, strains presenting rapeseed-meal-degrading and nonhemolysis were tested according to Didinen et al. [13] method. Bacterial culture (OD_600_ = 0.5) was inoculated with 30 μL aliquots into protease medium or cellulase medium. The protease medium was prepared as follows: solution A: 100 mL LB medium, 3% AGAR, sterilized at 121°C for 25 min; solution B: 1.5% skim milk powder 100 mL (Becton, Dickinson and Company, USA) sterilized at 115°C for 15 min, pH 7.0–7.2. Solutions A and B were mixed and poured into petri dishes. Cellulase medium: 100 mL LB medium, 1% sodium carboxymethyl cellulose (CMC-Na) (Guangzhou Meilun Biotechnology Co., Ltd), 1.5% AGAR, 1% KH_2_PO_4_, pH 7.0–7.2. Congo red (Beijing Solebao Technology Co., Ltd.) solution with distilled water was prepared with a concentration of 2000 mg/L, and stored in the dark. Congo red decolorizing solution: 1 mol/L NaCl.) for 24 h at 37°C. The diameter of the hydrolysis circle was measured.

### 2.4. Solid-State Fermentation

The rapeseed meal used in this study was purchased from Guangzhou Bolai Feed Co., Ltd; it was a commercially available product that had already been dried and crushed. Isolates presenting rapeseed-meal-degrading and nonhemolysis were incubated at 37°C for 12 h. The bacteria were harvested and adjusted to a concentration of 1.0 × 10^7^ CFU/mL using sterile water. Before inoculation, we calculated the amount of water required for 7 kg of rapeseed meal (wet basis) to reach the target moisture of 22.2% (w/w). Exactly this volume of the 1.0 × 10^7^ CFU mL suspension was then sprayed onto the meal and mixed thoroughly, so that the spray liquid always contained 10^7^ CFU mL and the final substrate moisture met the 22.2% specification without any additional water.

The inoculated meal (17 cm bed depth) was placed in a sterilized 48 L high-density polyethylene box. The lid was perforated with 20 3 mm holes to maintain aerobic conditions. Fermentation proceeded at ambient temperature (28 ± 1°C) for 48 h, with manual mixing every 12 h.

### 2.5. Strain Identification

#### 2.5.1. Biochemical Identification

The candidate strain was inoculated on LB agar at 37°C for 24 h. The shape, size, and color of the colonies were observed. The bacteria were examined post-Gram staining [14] using a light microscope (Nikon ECLIPSE E100). Additionally, In addition, spore staining was performed using a spore staining kit, and the percentage of spore-forming cells was determined by counting under a microscope.. The biochemical indexes were determined using API 50 CHB (bioMérieux, France).

#### 2.5.2. Molecular Identification

The genomic DNA of the candidate strain was extracted using a bacterial DNA extraction kit (TIANGEN BIOTECH [BEIJING] Co., Ltd, China), and the affinities of the candidate strain were identified using the 16S rRNA [15] and rpoB [16] genes. The rpoB primers were synthesized by Sangyo Bioengineering (Shanghai) Co. Reagent integrity was monitored by the intrinsic color shift of the Gram-stain solutions and by the manufacturer's built-in positive/negative indicators for each biochemical kit. No external reference strains were included; strain identity was instead verified by 16S rRNA sequencing and rpoB sequencing.

For 16S rRNA gene amplification, universal primers 27F (5′-AGAGTTTGATCCTGGCTCAG-3′) and 1492R (5′-GGTTACCTTGTTACGACTT-3′) were used. The PCR reaction system (25 μL) included 1 μL DNA template, 12.5 μL PCR Mix, 1 μL each of primers 27F and 1492R (10 μmol/L), and sterile water. PCR conditions: 94°C for 3 min (initial denaturation), followed by 34 cycles of 95°C for 30 s (denaturation), 55°C for 30 s (annealing), 72°C for 90s (extension), with a final extension at 72°C for 10 min.

For *rpoB* gene amplification, primers were synthesized by Sangon Biotech (Shanghai) Co., Ltd (F: 5′-AAAAGGTTTTACCGCAACTG-3′, R: 5′-CGCATCTTCTTCGTCTTCTA-3′). The PCR reaction system (25 μL) contained 1 μL DNA template, 12.5 μL PCR mix, 1 μL each of F and R primers (10 μmol/L), and sterile water. PCR conditions: 94°C for 3 min (initial denaturation), followed by 33 cycles of 95°C for 30 s (denaturation), 50°C for 30 s (annealing), 72°C for 120 s (extension), with a final extension at 72°C for 10 min.

PCR products (5 μL each) were analyzed using 1% agarose gel electrophoresis, and results were visualized with a gel imaging system. Positive PCR products, confirmed by electrophoresis, were sequenced by Aggie Biotechnology Co., Ltd, and the sequences were used for phylogenetic tree construction.

### 2.6. Drug Sensitivity Tests

The drug-sensitive paper diffusion method (K-B method) was used to determine the susceptibility of the candidate strain to common drugs according to the Executive Standards for Antimicrobial Susceptibility Testing of the Clinical and Laboratory Standards Institute (CLSI M100). The candidate strain was inoculated and cultured in liquid medium to an OD_600_ = 0.5. The bacterial solution was diluted 10-fold and dispensed onto MH solid medium. Postaffixing of antibiotic susceptibility disc, the plates were inverted and incubated at 37°C for 18–20 h. The diameter of the circle of inhibition of each antibiotic susceptibility disc was measured.

The minimum inhibitory concentration (MIC) was determined using a two-fold serial dilution method. First, 50 µL of LB liquid medium was added to each well of a 96-well plate. Next, 50 µL of the prepared antibiotic solution was added to the first column, followed by a two-fold serial dilution to generate a gradient of antibiotic concentrations. Finally, 50 µL of bacterial suspension was inoculated into each well, achieving a final bacterial concentration of 5 × 10^6^ CFU/mL and maintaining the antibiotic concentrations at 256, 128, 64, 32, 16, 8, 4, 2, 1, 0.5, 0.25, and 0.125 mg/L (prediluted to account for bacterial suspension addition). The positive control contained a bacterial solution without antibiotics, while the negative control consisted of antibiotics without bacterial inoculation. The MIC was defined as the lowest drug concentration that completely prevented visible bacterial growth in the culture tubes.

### 2.7. Pathogenicity Test

Grass carp with an average body weight of 30 ± 5 g and zebra fish with an average body weight of 2.5 ± 0.5 g were selected. Each type of fish was divided into three experimental groups and one control group, with 20 fish in each group. The fish were placed in a fish tank at 28°C for 1 week before the experiment.

The candidate strain was inoculated in LB broth medium at a ratio of 1:100, incubated at 37°C for 4 h. Then the bacteria was centrifuged (SIGMA 3K15) at 5000 rpm for 5 min, washed with saline and resuspended to obtain three bacterial concentrations of 1.5 × 10^9^, 1.5 × 10^8^, and 1.5 × 10^7^ cfu/mL. Fish were intraperitoneally challenged according to Zhang et al. [17]. The experiments were performed in triplicate. The challenged fish were monitored for mortality and morbidity for 7 days.

### 2.8. Whole Genome Sequencing Analysis

The candidate strain was inoculated and cultured in LB solid medium for 24 h. Thereafter, single colonies were picked, inoculated in LB broth medium, and cultured in a shaking incubator at 37°C for 12 h (180 rpm). The collected organisms were sent to Wuhan Fraser Genetic Information Co., Ltd. For whole gene sequencing using the third-generation PacBio sequencing technology.

#### 2.8.1. Comparative Genomic Analysis

Based on the sequencing results of the *rpoB* gene, four strains with a close genetic relationship to the candidate strain conserved protein-encoding gene *rpoB* were randomly selected. The sequence files of the *B. subtilis ATCC6633*, *KCTC13429*, *CCSR02*, and *DE111* genes were downloaded from the NCBI database for comparative genomic analysis with the candidate strain.

#### 2.8.2. Predictive Analysis of Resistance and Virulence Genes

Resistance genes in the whole genome of the candidate strain were predicted using the CARD online database and the following selection criteria: “perfect and strict match only.” A similarity of more than 80% and a coverage of more than 70% were used as the prediction criteria for the genes that may be present. The virulence genes of the candidate strains were predicted using the VFDB database, and the prediction criteria were consistent with those of drug resistance genes.

#### 2.8.3. Functional Gene Annotation

Metabolic function genes were annotated based on the genomic sequences of the candidate strain, and the selected databases included KEGG, COG, and CAZy.

#### 2.8.4. *bglA* Gene PCR Verification

##### 2.8.4.1. BSY82 Genomic DNA Extraction

Strain BSY82 was grown overnight in 100 mL of LB broth at 37°C with shaking (180 rpm). Genomic DNA was extracted with the TIANGEN Bacterial Genomic DNA Extraction Kit following the manufacturer's protocol, and the eluted DNA was collected in a microcentrifuge tube. The concentration and purity of the extracted DNA were assessed with a micro-volume spectrophotometer; acceptable samples showed an A260/A280 ratio between 1.7 and 1.9.

##### 2.8.4.2. Primer Design and PCR Amplification

Whole-genome annotation revealed an open reading frame (BSY82_1_03823) annotated as bglA, encoding a putative GH1 6-phospho-β-glucosidase. Gene-specific primers were designed with NCBI Primer-BLAST: bglA-F (5′- TTCCCAATTAAAGGAGGAAGGAT-3′) and bglA-R (5′-TTGTTCCAGGTAAGGTATAAAC-3′), targeting a conserved catalytic region. The bglA gene was amplified by PCR using genomic DNA of Bacillus subtilis strain BSY82 as template; the reaction composition is listed in Table 1. After the reagents were dispensed into an Eppendorf tube, the mixture was vortex-mixed and briefly centrifuged at 2000 r min−1 to collect the contents, and the sample was then subjected to PCR. Cycling parameters were as follows: 95°C for 5 min (initial denaturation); 33 cycles of 94°C for 30 s, 53°C for 30 s, and 72°C for 1.5 min; followed by a final extension at 72°C for 10 min and a hold at 4°C. An aliquot of 7.5 µL of the PCR product was electrophoresed on a 1% agarose gel at 120 V, and the amplicon size was assessed relative to the Marker 2000 DNA ladder.

### 2.9. Analysis of Myrosinase Activity

The myrosinase activity of strain BSY82 was determined using spectrophotometric and HPLC methods based on the hydrolysis of sinigrin (potassium myronate) to glucose. For the spectrophotometric assay, strain BSY82 was cultured in LB liquid medium at 37°C and 180 rpm for 24 h, followed by centrifugation at 8500 rpm for 10 min to collect the supernatant as crude enzyme. A 200 μL aliquot of the crude enzyme was mixed with 200 μL of 10 mg/mL sinigrin solution and incubated at 37°C for 20 min, after which 600 μL of color-developing reagent was added and boiled for 2 min. Absorbance was measured at 227 nm [18], and enzyme activity was calculated as the increase in glucose concentration per unit time (ΔGlucose/ΔT) [19], with a standard glucose curve prepared according to established protocols [20]. For the HPLC method, 200 μL of crude enzyme was diluted 5-fold, and 360 μL of the diluted enzyme was mixed with 40 μL of sinigrin solution, incubated at 37°C for 10 min, and terminated with 100 μL of concentrated HCl. The solution was filtered through a 0.22 μm membrane and analyzed using a Sepax C18 reversed-phase column (250 × 4.6 mm, 5 μm) with a mobile phase of 1% phosphoric acid in water and methanol (92.5:7.5) at a flow rate of 0.8 mL/min, column temperature of 30°C, detection wavelength of 235 nm, injection volume of 10 μL, and run time of 40 min. Myrosinase activity was defined as the product formation rate per unit substrate concentration.

### 2.10. ANFs Analysis

To determine the safety of the fermentation of rapeseed meal by the candidate strain, the contents of isothiocyanates (analyzed via gas chromatography (GC) according to GB/T 13087-2020) and oxazolidinethiones (determined by UV spectrophotometry as per GB/T 13089-2020) were measured [21]. Additionally, glucosinolates in the rapeseed meal were quantified using a modified spectrophotometric method described by Liu [22]. Fermented rapeseed meal was dried at 60°C, and 100 mg was weighed into a 10 mL tube. The sample was heated in a boiling water bath for 10 min, mixed with 8–10 mL of 90°C hot water, and incubated in boiling water for 30 min with intermittent mixing. After cooling, the solution was diluted to 10 mL with water and filtered. Test group: 0.5 mL filtrate was mixed with 2 mL of 0.15% CMC-Na and 1 mL of 8 μmol/L PdCl_2_ solution. The mixture was incubated at 20°C for 1 h, and absorbance (*E*_1_) was measured at 540 nm against a blank (distilled water). Control group: 0.5 mL filtrate was mixed with 2 mL of 0.15% CMC-Na and 1 mL of 0.03 mol/L HCl. Absorbance (*E*_2_) was measured similarly. Net absorbance (*E*) = *E*_1_–*E*_2_. Glucosinolate content (μmol/mL) was calculated as 0.2 + 185.2 × *E*.

The analysis was performed using GC under the following conditions: a polyethylene glycol (FFAP) capillary column (30 m length × 0.32 mm internal diameter, 0.25 μm film thickness) with a temperature program starting at 100°C (held for 10 min), ramping at 10°C/min to 200°C (held for 2 min). The injection port and detector temperatures were set to 220°C and 230°C, respectively, with a carrier gas (high-purity nitrogen) flow rate of 1.0 mL/min.

### 2.11. Statistical Analysis

The data was analyzed using one-way ANOVA to evaluate the phenotypic characteristics associated with candidate strains, including glucosinolates degradation rate, protease, and cellulase hydrolysis zone diameter. Multiple comparisons were conducted using the Waller–Duncan method to determine the significant differences between various treatments.

## 3. Results

### 3.1. Results of Strain Screening

**Figure 1:**
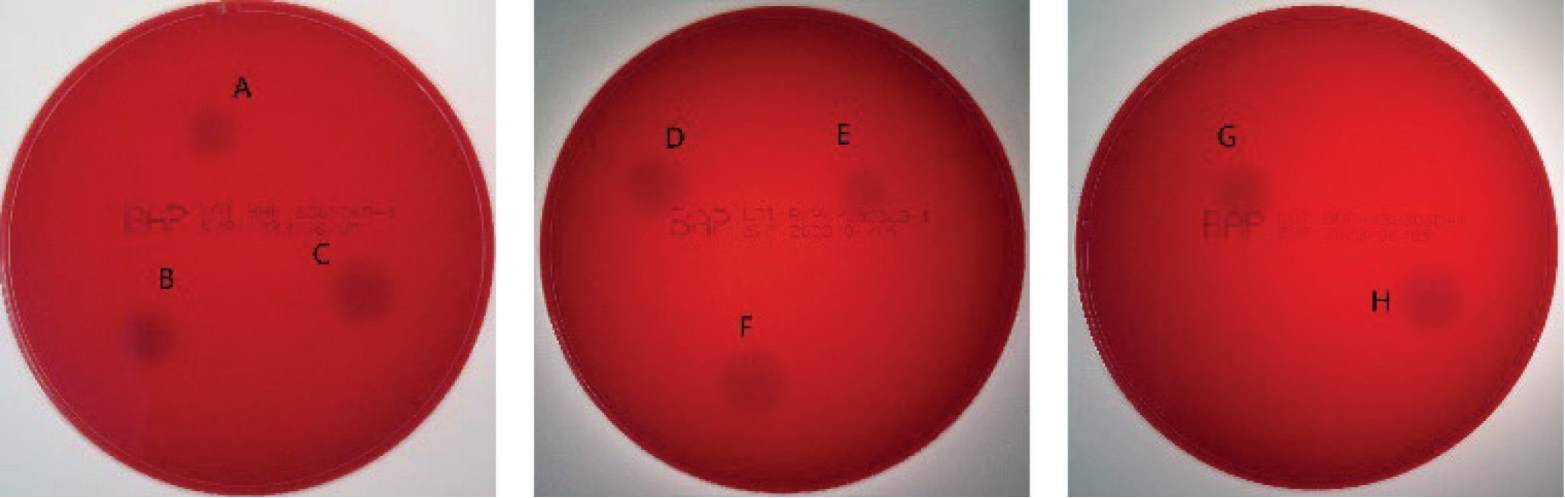
Hemolytic activity of candidate strains. (A) BSY84, (B) BSY101, (C) BL5, (D) BL35, (E) JD1, (F) BSY82, (G) DY85, and (H) BL126.

Eight isolates, BSY84, JD1, BSY101, BL35, BL126, DY85, BL35, and BSY82, with glucosinolate degradation ability and no hemolytic activity (Figure 1), were screened from 192 strains. Among the eight isolates, strain BSY82 exhibited the highest glucosinolate degradation efficiency (primary index) in rapeseed meal after solid-state fermentation, along with superior protease and cellulase production capacities, as summarized in Table 2. These activities support the strain's broader capacity to upgrade rapeseed-meal quality.

### 3.2. Identification of Isolate BSY82

#### 3.2.1. Morphological Identification

**Figure 2:**
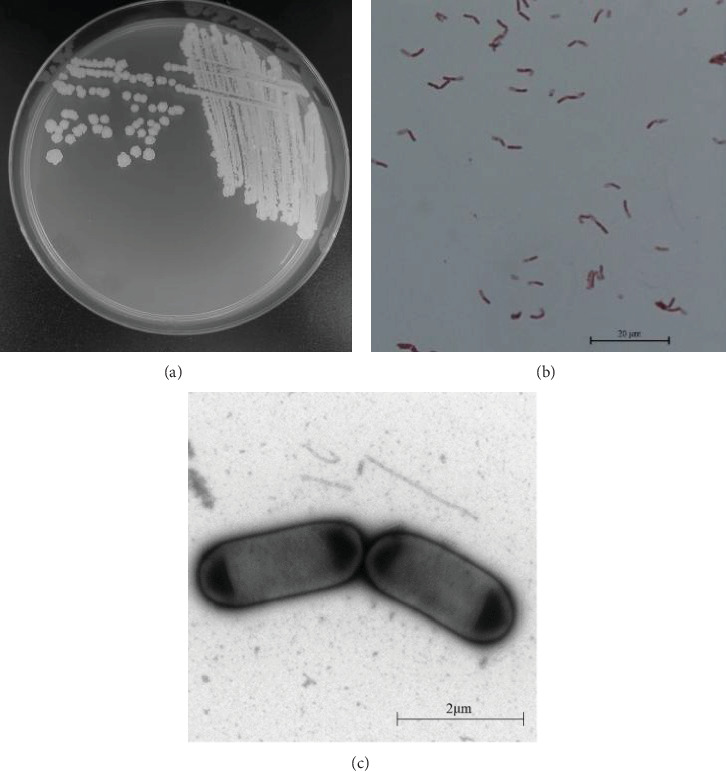
Morphology of BSY82 colonies and organisms. (A) Colonies, (B) Gram staining observation (×1000), and (C) observation of positive staining by projection electron microscopy (×1000000).

The colonies formed by the BSY82 isolate were round or oval, opaque, concave, folded on the surface, and irregular on the edge after being cultured on LB solid medium at 37°C for 24 h. The cells were short rods and stained purple as Gram-positive bacteria (Figure 2). The length of the bacterium was approximately 0.8 × 2.5 µm (width × length). The isolate was spore-forming, and the percentage of spores was 85%.

#### 3.2.2. Physiological and Biochemical Characterization

The physiological and biochemical results (Table 3), the isolate BSY82 could utilize a variety of carbohydrates, such as D-arabinose, ribose, glucose, fructose, etc., as carbon sources; beef paste, peptone, and yeast powder as organic nitrogen sources; and ammonium sulfate, ammonium chloride, ammonium citrate ammonium phosphate diammonium phosphate, etc., as inorganic nitrogen sources. A negative result was obtained in the oxidase assay, while a positive result was obtained in the peroxidase assay. With reference to the physiological and biochemical characteristics of the bacteria in the Bergey's Manual of Bacterial Identification [23], the isolate BSY82 was identified to belong to the genus *Bacillus*.

#### 3.2.3. Molecular Identification

**Figure 3:**
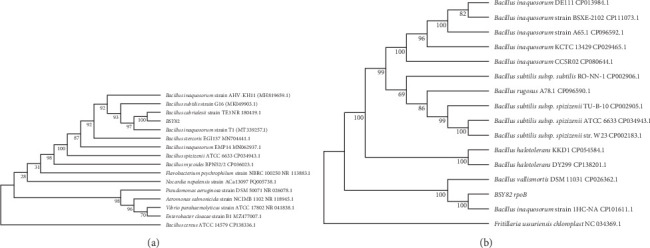
Phylogenetic tree of strain BSY82. (A) Phylogenetic tree constructed based on 16S rRNA gene and (B) phylogenetic tree constructed based on *rpoB* gene.

Sequencing analysis revealed that the 16S rRNA and *rpoB* genes amplified from strain BSY82 were 1547 and 2327 bp in length, respectively (NCBI accession number: CP126534.1). The neighbor-joining method was used to construct a phylogenetic tree based on the 16S rRNA and *rpoB* gene sequences. Both gene sequences of the isolate aggregate into a branch with *Bacillus subtilis subsp*. inaquosorum (Figure 3).

Based on the results, this strain BSY82 was a novel *Bacillus inaquosorum* strain capable of glucosinolate degradation during solid-state fermentation of the rapeseed meal [24].

### 3.3. Antibiotic Sensitivity Tests

The resistance of the strain BSY82 to common antibiotics was determined using the drug-sensitive paper diffusion method (Table 4). The strain was highly susceptible to 16 antibiotics, including roxithromycin, florfenicol, and amoxicillin, and moderately susceptible to penicillin and sulfisoxazole. The MICs of eight antibiotics required by the Guidelines for the Identification of Strains for the Production of Directly Fed Microorganisms and Fermented Products and their Safety Evaluation [25] were determined for strain BSY82. Based on the results, the strain was sensitive to vancomycin, gentamicin, kanamycin, streptomycin, erythromycin, clindamycin, and tetracycline, and resistant to chloramphenicol (Table 5). The animal pathogenicity assay revealed no mortality and adverse symptoms in grass carp and zebrafish at the three concentrations tested (1.5 × 10^7^, 1.5 × 10^8^, 1.5 × 10^9^), indicating that the BSY82 strain was not pathogenic to fish.

### 3.4. Assembly and Analysis of the Complete Genome

The whole genome of *B.inaquosorum* BSY82 consisted of one circular chromosome with a size of 4477503406 bp and a G + C content of 44.03%. A total of 4126 encoding genes were predicted, which accounted for 89% of the genome, with an average length of 890 bp.

#### 3.4.1. Drug Resistance Genes

Resistance genes are a class of genes present in bacteria or other microorganisms that enable microorganisms to resist the bactericidal effects of antibiotics, causing bacteria to develop antibiotic resistance [26]. By predicting the resistance genes in the whole genome of strain BSY82 using CARD online database (Table 6), BSY82 was recognized to potentially contain four resistance genes, *BSU-1*, *ykkC*, *ykkD*, and *mphK*. These genes may confer resistance to β-lactam, aminoglycoside, tetracycline, benzamides, and macrolide antibiotics via β-lactamase, antibiotic efflux pump, and macrolide antibiotic enzyme approaches, respectively. However, a discrepancy was found between this result and the results of the drug-sensitive paper slides, which may be due to the fact that the resistance genes are not expressed or the expression is not sufficiently low to provide sufficient resistance. Gene prediction showed low gene similarity, although strain BSY82 was found to have the chloramphenicol resistance gene *catA*, which inactivates chloramphenicol by acetylating it through encoding chloramphenicol acetyltransferase [27]. Several genes are intrinsic resistance genes of the BSY82 strain, and gene prediction did not find the presence of plasmids and transposons in the strain, reducing the risk of horizontal transfer.

#### 3.4.2. Virulence Genes

Virulence gene prediction for strain BSY82 was performed using the VFDB database (Table 7). A total of 19 potential virulence genes were identified; however, functional annotation revealed that the encoded proteins were primarily associated with bacterial growth and reproduction. No known *Bacillus* virulence factors, such as the hemolysin BL gene (*hblC*), enterotoxin T gene (*bceT*), cytotoxin K gene (*cytK*), pleiotropic regulatory factor (*plcR*), or nonhemolytic enterotoxin gene (*nheA*), were detected. These findings suggest that strain BSY82 lacks pathogenic potential, which is consistent with the results of animal pathogenicity experiments.

#### 3.4.3. Functional Genes

The whole genome sequence of the BSY82 strain was functionally annotated using COG, KEGG, and CAZY databases. A total of 3009 genes were annotated in the COG database, with a total of 20 major categories, primarily transcription, amino acid metabolism, and carbohydrate transport and metabolism, respectively. A total of 2470 genes were annotated based on KEGG, corresponding to the KEGG pathways, which contain six major categories, namely cellular processes, metabolism, organic systems, genetic information processing, human diseases, and environmental information processing, with carbohydrate metabolism and amino acid metabolism as the most important. A total of 100 glycoside hydrolase genes, which are mainly from the glycoside hydrolase family, were annotated in the CAZY database. GH1, GH13, GH30, GH43, GH109, and GH117. Some of the annotation results were unified (Table 8). Among the annotated genes, the *bglA* gene was identified. Previous studies have reported that this gene encodes a 6-phospho-β-glucosidase (BSY82_1_03823) exhibiting myrosinase activity [28, 29]. Translating the nucleotide sequence of the *bglA* gene from the whole genome into its corresponding protein showed an 87.61% similarity (*e*-value 0.0) with the reference sequence of 6-phospho-β-glucosidase (*bglA*) from *Bacillus amyloliquefaciens* (accession: WP_064107630.1). BLASTp comparisons against known microorganisms possessing confirmed myrosinase activity showed high similarity with several strains: 86.86% similarity (*e*-value 2e–116) with the *bglA* sequence from *Bacillus sp*. NGB-B10 (accession: BDH43810), 64.78% similarity (*e*-value 0.0) with the *bglA* sequence from *Enterobacter ludwigii* strain EcWSU1 (accession: AEW75128), 51.55% similarity (*e*-value 0.0) with the *ascB* sequence from *Escherichia coli* O104:H4 (accession: EGT69672), and 65.20% similarity (*e*-value 0.0) with the *bglA* sequences from *E. coli* O157:H7 strains TW14359 (accession: ACT73612) and Sakai (accession: BAB37196). Additionally, it showed 26.94% similarity (*e*-value 4e–49) with plant myrosinase from *Brassica juncea* (accession: AAG54074). However, no significant sequence similarity was found when compared to the myrosinase from *Citrobacter sp*. strain WYE1 (accession: KT821094.1).

#### 3.4.4. PCR Verification of *bglA* Gene

**Figure 4:**
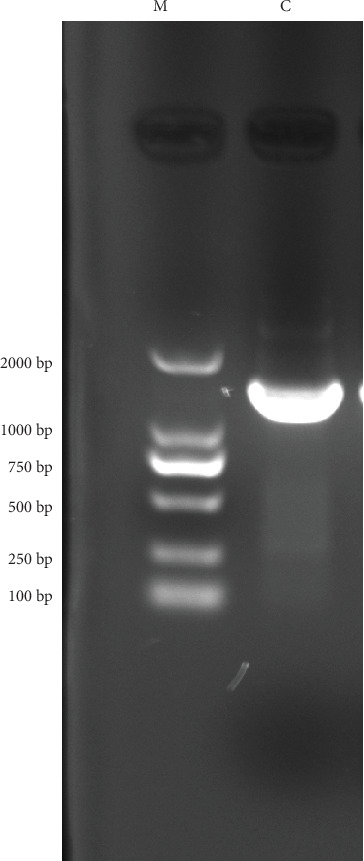
Electrophoresis of *bglA* gene amplification by PCR gene fragment M: DL2000 marker C: target.

After electrophoresis on a 1% agarose gel, the PCR product produced a single band of approximately 1600 bp (Figure 4), consistent with the expected fragment size.

### 3.5. Strain Myrosinase Activity

**Figure 5:**
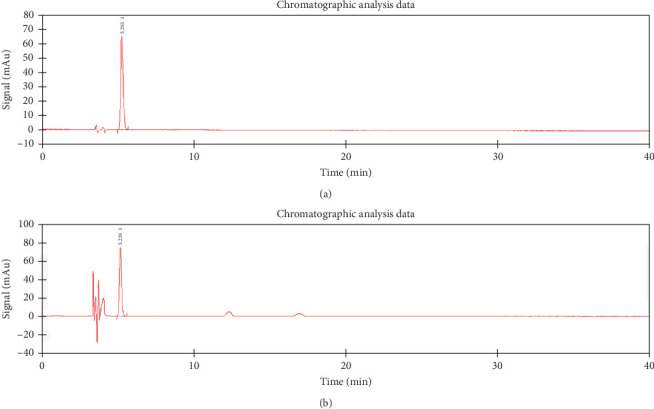
Activity profile of myrosinase. (a) Shows the standard profile of myrosinase with a peak area of 1032.317 and (b) shows the activity profile of myrosinase in the bacterial solution with a peak area of 870.9460.

The fermentation broth of the BSY82 strain was used as the crude enzyme solution. The glucosinolate content in the crude extract was reduced by 68% after incubation of the crude enzyme solution with the glucosinolate crude extract for 1 h. A spectrophotometric measurement at 227 nm gave an absorbance of 2.72, which corresponds to a myrosinase activity of 1.67 µmol min according to the conventional activity definition. This chromogenic method provides a rapid, semi-quantitative estimate of overall glucosinolate degradation but can underestimate activity when pigments or insoluble particles partially mask the color signal. To obtain a substrate-specific and highly accurate value, we next quantified the disappearance of pure sinigrin by HPLC. Enzyme activity = product generation rate/substrate concentration. Under the optimized chromatographic conditions described in Section 2.9, the crude enzyme hydrolyzed sinigrin at a rate of 2.12 ± 0.068 µmol min (Figure 5). These complementary assays demonstrate that strain BSY82 possesses robust myrosinase activity, with the HPLC method providing a precise rate for a defined glucosinolate substrate.

### 3.6. ANFs in Fermented Rapeseed Meal

The experimental results (Table 9) revealed that the glucosinolate content of the rapeseed meal reduced from 23.91 to 7.8 μmol/g, with a degradation rate of 67.38%. The isothiocyanate content reduced from 46 to 21.03 mg/kg, with a degradation rate of 54.28%. Oxazolidinethione was not detected at 0 and 48 h of the fermentation.

## 4. Discussion

In the present study, the strain BSY82, which lacks hemolytic activity but exhibits glucosinolate degradation ability, was screened from 192 bacterial strains. Based on morphological, physiological, biochemical, 16S rRNA, and *rpoB* genetic analyses, this strain was identified as *Bacillus subtilis subsp*. *iniquosorum*, a Desert subspecies of *Bacillus subtilis*.

As an aquatic probiotic, the addition of probiotics to aquaculture water can improve the water quality, improve animal immunity, and inhibit many pathogenic microorganisms [30–33]. The addition of probiotics to feed can improve the intestinal flora of animals, thereby enhancing the immunity of animals and promoting growth. Several species of *Bacillus* exist. However, only two species, *Bacillus subtilis* and *Bacillus licheniformis*, can be used in feed production [34]. The hemolytic and animal pathogenicity assays revealed that the strain BSY82 lacked hemolytic activity, and animals did not die or experience undesirable symptoms during the experimental observation period. The prediction of virulence genes revealed that the strain BSY82 comprised the *clpC*, *clpP*, *tufA*, *groEL*, *dhbE*, and *sigA* genes, which were mainly related to the basic physiological functions of the bacterium, such as growth, spore production, biofilm formation, protein synthesis, and functions such as DNA repair and transcription [35–40]. Virulence factors can influence microbial invasion of the host, evasion of the host immune system, destruction of host tissues, or modulation of the host immune response [41]. Virulence factors can include bacterial exotoxins, endotoxins, bacterial attachment factors, bacterial secretion systems, bacterial flagella and cilia, and others. Owing to these factors, microorganisms are better able to cause infection and lead to disease development [42]. The presence of hemolysin genes (*hblA*, *hblC*, and *hblD*), nonhemolytic enterotoxin (*Nhe*), cytotoxin (*CytK*), and other virulence genes known to be pathogenic was not detected in the virulence gene prediction of this strain, which revealed the high biosafety of the strain BSY82 at the genotype and phenotype levels.

Bacterial resistance refers to the ability of bacteria to resist antibiotics or other drugs. Owing to the extensive use of antibiotics, some bacteria have acquired resistance genes through gene mutation or horizontal gene transfer and have thus become resistant. At present, the resistance genes of *B. cereus* are mainly acquired through horizontal gene transfer, and few reports have been published on the inherent resistance basis of *B. cereus* [43]. In this study, we screened and obtained a number of *Bacillus subtilis* resistance genes. The resistance results of *B. iniquosorum* BSY82 revealed that the strain was sensitive to common antibiotics and resistant to chloramphenicol. The resistance gene prediction results revealed that the strain had *BSU-1*, *ykkC*, *ykkD*, and *mphK* resistance genes, which might be resistant to β-lactam, aminoglycosides, tetracyclines, benzylamides, and macrolides. The resistance gene prediction results differed from those of the resistance experiments. Thus, the strain might lack appropriate transcription factors or other regulatory factors, which may prevent the activation and expression of the resistance genes. In addition, the expression of the resistance genes may be insufficient to produce resistance to the corresponding antibiotics. In the MIC experiments, the strain BSY82 was resistant to chloramphenicol, and no chloramphenicol efflux pumps were found in the results of the resistance gene prediction for the gene, *cmlA* [44]. Moreover, the strain did not contain a plasmid, suggesting that chloramphenicol resistance in this strain was not acquired by exocytosis pump discharge and gene-level transfer. The similarity of the chloramphenicol acetyltransferase gene, *catA*, in the gene prediction results was only 58.33%, indicating a low gene similarity. Thus, whether the chloramphenicol resistance of this strain is determined by the catA gene is uncertain.

*Bacillus subtilis* is often used as a fermentation strain in feed fermentation owing to its high biosafety. As a common feed ingredient, rapeseed meal is used in the fermentation and detoxification of *Bacillus subtilis*. The ANFs in rapeseed meals are mainly glucosinolate and their hydrolysis products. Glucosinolate is nontoxic; however, the rapeseed meal structure is destroyed during processing, and glucosinolate reacts with myrosinase to produce toxic substances (oxazolidinedione and isothiocyanate), which can be detoxified by four main routes, namely thermal degradation, chemical degradation, microbial degradation, and enzymatic degradation [45–47]. Thermal degradation can reduce the toxicity of glucosinolate by blunting myrosinase activity. Chemical degradation involves destroying the chemical structure of glucosinolate using an acid or alkali, enabling glucosinolate to produce oxazolidinethione and isothiocyanate, and then volatilizing these products at high temperatures to achieve detoxification [48]. Microbial degradation of glucosinolate mainly occurs via the secretion of isozymes that have the same functional activities as those of myrosinase, which was previously thought to be produced by plants, insects, and fungi only [49]. Previously, myrosinase was thought to be produced by plants, insects, and fungi alone; however, bacteria, such as *Citrobacter* Wye1, *Sphingobacterium sphaericus* RJ35, *Bacillus sphaericus* NGB-B10, etc., can also produce myrosinase [39–41]. Microbial degradation is nonpolluting and cost-effective. Moreover, microbial degradation has the advantages of nonpollution, low cost, and high efficiency; thus, microbial degradation is mainly used in rapeseed meal detoxification. In this study, a strain of *Bacillus subtilis*, BSY82, was screened. The content of glucosinolate was reduced by 67% after solid-state fermentation of the rapeseed meal using this strain. However, the content of isothiocyanates was reduced by 54%. This result indicates that the content of glucosinolate was reduced after fermentation of the strain BSY82, and glucosinolate was not only not decomposed into isothiocyanate and oxazolidinethione, but also the content of isothiocyanate in the meal was reduced, and the nutritive value of the rapeseed meal was improved [50]. The observed 68% reduction in glucosinolate content after 1 h of incubation with the crude enzyme solution demonstrates that strain BSY82 effectively degrades glucosinolates through extracellular enzymatic activity. This capability aligns with its potential role in rapeseed meal detoxification, where hydrolysis of glucosinolates by microbial enzymes is critical for reducing antinutritional compounds. This study also observed that the BSY82 strain has protease and cellulase activities, indicating that this strain can not only remove glucosinolates but also degrade crude fiber and macromolecular proteins in rapeseed meal, improving the nutritional value of rapeseed meal. Future work will correlate these enzymatic activities with changes in protein, peptide, and neutral-detergent fiber contents of the fermented product. Meanwhile, myrosinase activity was detected in the fermentation broth of strain BSY82, with an enzyme activity of 2.12 ± 0.068 U and 1.67. The difference in numerical values between the two assays is expected: the colorimetric test measures total glucosinolates in a complex matrix, whereas the HPLC assay monitors a single soluble glucosinolate under optimal conditions. Together, the complementary methods confirm robust myrosinase activity in strain BSY82. The reported enzyme activity of Myr-37 [51] was 6.95 U, suggesting that strain BSY82 can degrade glucosinolate by producing an enzyme system with a similar function to myrosinase. All myrosinases in bacteria are isoenzymes of plant myrosinases, and both belong to the glycoside hydrolase family. In this study, the whole genome sequencing results of the BSY82 strain based on CAZY predicted numerous glycoside hydrolase genes, which should be explored and analyzed to identify the myrosinase genes in this strain.

## 5. Conclusion

In this study, an isolate (BSY82) with glucosinolate degradation ability was screened among the 192 isolated bacteria from the Grass carp culture pond of Pearl River Fisheries Research Institute. The fermentation broth of this strain exhibits myrosinase activity, and the glucosinolate degradation rate of the fermented and meal treated with the BSY82 strain was as high as 67%. BSY82 belongs to the glucosinolate-degrading strains and has high efficiency. The BSY82 strain is sensitive to common antibiotics and is not pathogenic to experimental animals. The safety of this strain was verified at the genetic level, which revealed high safety. It complies with the relevant standards of the “Guidelines for the Identification of Strains for the Production of Directly Fed Microorganisms and Fermented Products and their Safety Evaluation” designated by the Ministry of Agriculture and Rural Affairs [25]. The BSY82 strain can be used as a high-quality fermentation strain of rapeseed meal, thereby increasing the value of rapeseed meal, and enabling the “action of reducing and replacing the amount of soybean meal.”

## Figures and Tables

**Table 1:** PCR amplification system of the *bglA* gene.

Component	Volume
2× premix taq (taq version)	12.5 µL
Forward primer F (10 pmol L⁻^1^)	1 µL
Reverse primer R (10 pmol L⁻^1^)	1 µL
Template DNA	2 µL
DEPC-treated water	8.5 µL

**Table 2:** Glucosinolate degradation rate, protease, and cellulase hydrolysis circle diameter.

Code	Source	Rapeseed meal hydrolysis circle (mm)	Ratio of diameter of Rapeseed meal hydrolysis and colonies H/D	Protease hydrolysis circle (mm)	Ratio of diameter of Protease hydrolysis and colonies H/D	Cellulase hydrolysis circle (mm)	Ratio of diameter of Cellulase hydrolysis and colonies H/D	Glucosinolate degradation rate (%)
BSY82	Water	37.59	5.37 ± 0.59a	27.73	3.96 ± 0.77a	19.20	2.74 ± 0.17ad	67.54 ± 0.34a
BSY84	Grass carp	36.37	5.20 ± 0.42bd	24.47	3.50 ± 0.33bc	18.43	2.63 ± 0.12b	64.33 ± 0.31b
BSY101	Grass carp	32.46	4.64 ± 0.49c	27.70	3.96 ± 0.75a	19.03	2.72 ± 0.13ad	58.96 ± 0.18c
BL35	Water	36.77	5.25 ± 0.37b	23.64	3.38 ± 0.41c	13.03	1.86 ± 0.18c	64.37 ± 0.54b
BL126	Grass carp	30.25	4.32 ± 0.23e	24.55	3.50 ± 0.44bc	19.30	2.76 ± 0.06d	53.42 ± 0.24d
JD1	Grass carp	31.91	4.56 ± 0.23c	27.89	3.98 ± 0.75a	19.73	2.82 ± 0.23d	54.25 ± 0.71e
BL5	Water	28.61	4.09 ± 0.52f	25.25	3.61 ± 0.33b	18.90	2.7 ± 0.29ab	49.63 ± 0.34f
DY85	Water	35.77	5.11 ± 0.58d	21.33	3.05 ± 0.31d	12.30	1.76 ± 0.46e	63.57 ± 0.30g

*Note:* The data are presented as mean ± SD. Mean values in the same column with same letters are not significantly different (*p* > 0.05), whereas different letters within a column represent a significant difference (*p* < 0.05).

**Table 3:** Physiological and biochemical identification results of strain BSY82.

Identification of the item (substrate)	Positive (+)/negative (−)	Identification of the item (substrate)	Positive (+)/negative (−)
Blank control	−	Heptaphyllum esculin	+
Glycerol	−	Salicin	+
Erythritol	−	D-cellobiose	+
D-arabinose	−	D-maltose	+
L-arabinose	+	Lactose D-lactose	−
Ribose D-ribose	+	Melibiose	−
D-xylose	+	Sucrose D-saccharose	+
L-xylose	−	D-trehalose	+
Adonitol	−	Inulin	+
Methyl-beta-D-xylopyranoside	−	Melezitose	−
Galactose D-galactose	−	Cotton seed sugar D-raffinose	+
D-glucose	+	Starch amidon	+
D-fructose	+	Glycogen	+
D-mannose	+	Xylitol	−
Sorbose	−	Gentiobiose	+
L-rhamnose	−	D-turanose	−
Spearmintol dulcite	−	D-lyxopyranose	−
Inositol	−	D-tagatose D-tagatose	−
D-mannitol	+	D-fucose	−
Sorbitol D-sorbitol	+	L-fucose	−
α-methyl-D-mannoside	−	D-arabinitol D-arabitol	−
α-methyl-D-glucoside	−	L-arabitol	−
N-acetylglucosamine	+	Potassium gluconate	−
Amygdalin	+	Potassium 2-ketogluconate	−
Arbutin	+	Potassium 5-ketogluconate	−
Oxidase	−	Catalase	+

**Table 4:** Drug sensitivity tests of strain BSY82.

Antibiotics	Judgment standard (mm)				
	Insensitive (*R*)	Moderately sensitive (*I*)	Highly sensitive (*S*)	Inhibitory circle diameter (mm)	Sensitivities
Roxithromycin	<15	16–20	>21	25.2 ± 0.72	*S*
Florfenicol	<17	18–20	>21	26.1 ± 0.3	*S*
Amoxicillin	<13	14–17	>18	23.8 ± 1.15	*S*
Fleroxacin	<15	16–18	>19	29 ± 2.5	*S*
Cefadroxil cephalexin	<14	15–17	>18	31.7 ± 2.69	*S*
Penicillin	<19	20–27	>28	27.3 ± 0.78	*I*
Doxycycline	<12	13–15	>16	28.4 ± 0.36	*S*
Norfloxacin	<12	13–16	>17	29.9 ± 1.74	*S*
Ofloxacin	<12	13–15	>16	32.9 ± 0.44	*S*
Streptomycin	<11	12–14	>15	20 ± 0.26	*S*
Enrofloxacin	<15	16–20	>21	29.1 ± 0.4	*S*
Vancomycin	<14	15–16	>17	18.3 ± 0.36	*S*
Sulfisoxazole	<12	13–16	>17	15.9 ± 0.1	*I*
Spectinomycin	<14	15–17	>18	18 ± 0.26	*S*
Trimethoprim	<10	11–16	>17	23.5 ± 0.56	*S*
Tobramycin	<12	13–14	>15	21.7 ± 1.01	*S*
Erythromycin	<13	14–22	>23	23.2 ± 0.69	*S*
Gentamicin	<12	13–14	>15	21.2 ± 0.62	*S*

**Table 5:** MIC (minimum inhibitory concentration) experiment of strain BSY82.

Antibiotics	Threshold (mg/L)	MIC value (mg/L)	Sensitivity (sensitive/resistant)
Vancomycin	4	0.5	Sensitive
Gentamicin	4	0.25	Sensitive
Kanamycin	8	1	Sensitive
Streptomycin	8	1	Sensitive
Erythromycin	4	<0.125	Sensitive
Clindamycin	4	4	Sensitive
Chloramphenicol	8	16	Drug-resistant
Tetracycline	8	<0.125	Sensitive

**Table 6:** Predicted resistance genes in strain BSY82.

Gene name	Types of antibiotics	Function	Similarity (%)	Coverage (%)
*BSU-1*	Beta-lactam	Antibiotic inactivation	99.25	100
*ykkC*	Aminoglycosides, tetracyclines, and benzamides	Antibiotic discharge pumps	96.19	100
*ykkD*	Aminoglycoside, tetracycline, and benzamide antibiotics	Antibiotic discharge pumps	92.86	100
*mphK*	Macrolide antibiotic	Antibiotic inactivation	80.72	100
*catA*	Chloromycetin	Antibiotic inactivation	58.33	100

**Table 7:** Annotation results from VFDB database.

Gene name	Gene function	Similarity (%)
*clpC*	Endopeptidase *clp* ATP-binding chain C	78.73
*clpP*	ATP-dependent *clp* protease proteolytic subunit	78.42
*tufa*	Elongation factor *tu*	75
*groEL*	Chaperonin *groEL*	73.96
dhbE	2,3-dihydroxybenzoate adenylase	72.66
*sigA*/*rpoV*	RNA polymerase sigma factor *sigA*	71.25
*gndA*	NADP-dependent phosphogluconate dehydrogenase	70.20

**Table 8:** Annotation results from COG, KEGG, and CAZY databases.

Comprehensive database	Functional annotation results	Quantities
COG	Amino acid transport and metabolism	322
	Carbohydrate transport and metabolism	240
	Transcription	250

KEGG	Cellular processes	151
	Environmental information processing	329
	Genetic information processing	213
	Human diseases	53
	Metabolism	1698
	Organismal systems	26

CAZY	Glycoside hydrolases	100
	Glycosyl transferases	104
	Polysaccharide lyases	11
	Carbohydrate esterases	55
	Auxiliary activities	33
	Carbohydrate-binding modules	21

**Table 9:** Contents of antinutritional factors after 48 h of fermentation.

Fermentation time (h)	Glucosinolate (μmol/g)	Isothiocyanate (mg/kg)	Oxazolidinethione (mg/kg)
0	23.91	46.00	Not detected (limit 150 mg/kg)
48	7.80	21.03	Not detected (limit 150 mg/kg)

## Data Availability

The data that support the findings of this study are available from the corresponding author upon reasonable request.
